# Cognitive functions of regularly cycling women may differ throughout the month, depending on sex hormone status; a possible explanation to conflicting results of studies of ADHD in females

**DOI:** 10.3389/fnhum.2014.00191

**Published:** 2014-04-01

**Authors:** Ronit Haimov-Kochman, Itai Berger

**Affiliations:** ^1^Unit of Reproductive Endocrinology and Infertility, Department of Obstetrics and Gynecology, Hadassah Hebrew University Medical CenterJerusalem, Israel; ^2^The Neuro-Cognitive Center, Pediatric Wing, Hadassah Hebrew University Medical CenterJerusalem, Israel

**Keywords:** menstrual cycle, sex hormones, cognitive functions, attention deficit/hyperactivity disorder, gender

## Abstract

Attention Deficit/Hyperactivity Disorder (ADHD) is considered as a model of neuro-developmental cognitive function. ADHD research previously studied mainly males. A major biological distinction between the genders is the presence of a menstrual cycle, which is associated with variations in sex steroid hormone levels. There is a growing body of literature showing that sex hormones have the ability to regulate intracellular signaling systems that are thought to be abnormal in ADHD. Thus, it is conceivable to believe that this functional interaction between sex hormones and molecules involved with synaptic plasticity and neurotransmitter systems may be associated with some of the clinical characteristics of women with ADHD. In spite of the impact of sex hormones on major neurotransmitter systems of the brain in a variety of clinical settings, the menstrual cycle is usually entered to statistical analyses as a nuisance or controlled for by only testing male samples. Evaluation of brain structure, function and chemistry over the course of the menstrual cycle as well as across the lifespan of women (premenarche, puberty, cycling period, premenopause, postmenopause) is critical to understanding sex differences in both normal and aberrant mental function and behavior. The studies of ADHD in females suggest confusing and non-consistent conclusions. None of these studies examined the possible relationship between phase of the menstrual cycle, sex hormones levels and ADHD symptoms. The menstrual cycle should therefore be taken into consideration in future studies in the neurocognitive field since it offers a unique opportunity to understand whether and how subtle fluctuations of sex hormones and specific combinations of sex hormones influence neuronal circuits implicated in the cognitive regulation of emotional processing. The investigation of biological models involving the role of estrogen, progesterone, and other sex steroids has the potential to generate new and improved diagnostic and treatment strategies that could change the course of cognitive-behavioral disorders such as ADHD.

## Introduction

Behavioral, biochemical, and physiological data in animals demonstrate that gonadal steroid hormones estrogen, progesterone and testosterone have an effect on behavior and modulate neuronal activity (McEwen and Alves, 1999; Pfaff et al., 2000; McEwen, 2002; Pfaff, 2005). These hormones not only influence ovulation and reproductive behavior but may also have an effect on cognitive functions, affective state, vulnerability to drugs of abuse, and pain sensitivity (Bromberger and Kravitz, 2011; McEwen et al., 2012). While the influence of sex hormones on emotional states is supported by a wide body of animal data and reflected in diverging prevalence rates for men and women for psychiatric diseases, much too little is known about the impact of estrogen progesterone and testosterone on cognitive functions in women (Schmidt et al., 1998; Bloch et al., 2000). Common psychiatric disorders in women, such as depression and anxiety (Soares and Zitek, 2008) are associated with cognitive biases to emotional information. Furthermore, hormonal transitions across women’s lifespan, such as the premenstrual period (Rapkin and Akopians, 2012), postpartum (O’Hara, 2009) and menopause (Freeman, 2010) have been shown to be highly vulnerable to mood disorders, whereas alterations in the cognitive function during these periods were little investigated. The menstrual cycle offers a unique opportunity to study whether and how subtle fluctuations of sex hormones can influence cognitive functions.

### The menstrual cycle and hormonal profiles during women’s life span

**Figure 1:**
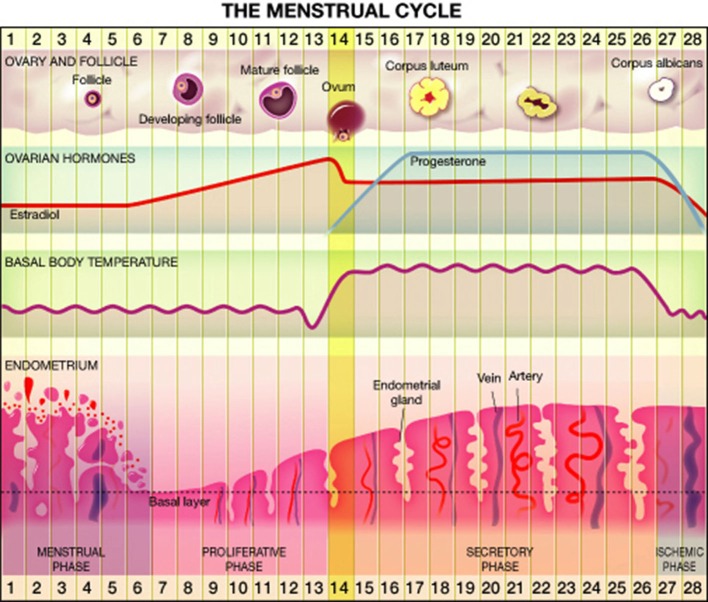
**The menstrual cycle**.

Most women (80%) experience regular menstrual cycle from menarche to menopause, whereas the rest have irregular cycles (Münster et al., 1992). Overall, approximately 15% of reproductive age cycles are 28 days in length (Vollman, 1997) divided into follicular and luteal phases. Significant changes in hormonal levels occur during the menstrual cycle. At the early follicular phase levels of estrogen, progesterone and testosterone are very low, while toward the mid-follicular days blood levels of estrogen and testosterone begin to rise, reaching maximal levels a little before ovulation (Griffin and Ojeda, 2004; Terner and De Wit, 2006). The rise in estrogen level is accompanied by a drop in follicle stimulating hormone (FSH) level. Ovulation occurs 40–44 h after a luteinizing hormone surge and a milder FSH surge. The luteal phase is characterized by moderate estrogen and increasing progesterone production by the corpus luteum. Estrogen decreases from moderate level at the midluteal phase to its lowest level just before the onset of menstruation. Progesterone levels rise after ovulation, peak approximately 7 days post ovulation on and fall rapidly just before menstruation to undetected levels (Griffin and Ojeda, 2004; Terner and De Wit, 2006). Details are presented in Figure 1.

Before menarche and after menopause estrogen and progesterone levels are usually un-measurable. In premenopausal years, and depletion of the follicular reserve of the ovary, the cycle length tends to shorten, and anovulatory cycles are more frequent, until its cessation during menopause.

### Impact of sex hormones on brain structure and function

It has been established that sex hormones act on the central nervous system and influence the organization of neural circuits during the prenatal period (Collaer and Hines, 1995; McEwen, 2001). While men have greater overall brain volume than women, relative to total volume, sex-specific regional differences exist. Men have larger amygdala and hypothalamus, while women have larger caudate and hippocampus. These regional differences may be related to the distribution of estrogen (hippocampus) and androgen (amygdala) receptors. Sex hormones are known to directly influence the hypothalamus and the hippocampus: areas that are implicated in emotional processing, perception and memory, as well as in the interpretation of sensory information (Fanselow and Dong, 2010; Hines, 2010).

As it becomes clearer that hormonal transition periods across the life span of women also affect brain organization, some newly neuroimaging studies have started addressing the relevance of subtle hormonal fluctuations across the menstrual cycle on brain architecture, connectivity, metabolism and blood flow. For example, there is some evidence that estrogen in postmenopausal women increases regional cerebral blood flow (Resnick et al., 1998; Maki and Resnick, 2000; Kaya et al., 2008), thus estrogen may account for some of the variability in blood flow and metabolism in women’s brain. Regional cerebral metabolic rate of glucose (CMRglu) varies significantly with menstrual cycle phase suggesting that there are acute hormonal effects on brain glucose metabolism (Reiman et al., 1996).

Genetic and hormonal differences are the two most obvious possible causes for gender differences in neuro-cognitive-behavioral aspects (Mahone, 2010). Sex steroids are major modulators of mammalian brain function, regulating neurotransmitters and influencing neuronal differentiation, growth, and synapse formation (Miodovnik et al., 2012). Exposure to varying levels of sex steroids early in development can lead to permanent changes in behavior (Morris et al., 2004). Sex hormones were found in a variety of clinical settings to impact major neurotransmitter systems of the brain. Women tend to have more active serotonin (5-HT transporter, 5-HT1A and 5-HT2A receptors) (Fink et al., 1998), dopamine (DA transporter) and GABA (neurotransmitter levels) systems. Estrogen and progesterone are involved in several aspects of brain function, such as brain development, synaptic plasticity, and modulation of neurotransmitter systems [e.g., serotonin, norepinephrine, γ-aminobutyric acid (GABA), glutamate] (Rubinow and Schmidt, 2006). Estrogen and progesterone receptors are found in brain areas involved with the stress response and mood regulation including the hypothalamus, hippocampus, amygdala, and prefrontal cortex (Lokuge et al., 2010; Bromberger and Kravitz, 2011).

5-HT functions to coordinate complex sensory and motor patterns during a variety of behavioral states and is implicated in the pathology of mood disorders, sleep and eating disorders, and schizophrenia. There is an association between estrogen and schizophrenia. A deficiency in estrogen exposure may impact gray matter cortical thickness, which may be reversed by higher levels of estrogen that may induce or activate neuroprotective mechanisms (van der Leeuw et al., 2013). The results of this and other studies fit both the estrogen deficiency and protection hypothesis (Begemann et al., 2012; McEwen et al., 2012).

Interestingly, studies on the effects of exogenous sex steroids in postmenopausal women have demonstrated higher 5HT2A binding throughout the cerebral cortex in women treated with estradiol plus progesterone replacement (Moses et al., 2000). Dopaminergic function is also enhanced in women. DA is important for reward processes including the reinforcing effects of most drugs of abuse, and has been implicated in a variety of neuropsychiatric disorders including Parkinson’s disease. The DA transporter, which functions to regulate synaptic DA availability, is higher in women compared to men (Lavalaye et al., 2000; Mozley et al., 2001; Staley et al., 2001). Healthy women may have higher presynaptic dopaminergic tone in striatum and higher extrastriatal DA receptor density and availability compared to men (Kaasinen et al., 2001; Laakso et al., 2002). The availability of D2 receptor may vary with fluctuations in sex steroid hormones across the menstrual cycle (Wong et al., 1988).

Although not as well studied, differences between men and women have been reported for other receptor systems. These include the cholinergic system, which is involved in memory and cognition; the GABAergic system, the major inhibitory neurotransmitter system involved in mood and memory; and the opioid system, which is involved in pain and reward processes. Women express higher numbers of cortical muscarinic acetylcholine receptors (Yoshida et al., 2000). Women have also higher cortical GABA levels than men as measured with magnetic resonance spectroscopy (MRS; Sanacora et al., 1999). GABA levels vary across the menstrual cycle (Epperson et al., 2002) such that cortical GABA levels declines between the follicular and luteal phase in healthy women, and increases in women with premenopausal dysphoric syndrome. This indicates that GABA neurotransmission in tightly regulated by the menstrual cycle.

### Sex differences on brain cognitive performance

Women are believed to have better verbal skills and inferior spatial abilities than men. In women, IQ correlates with gray matter volume of the frontal lobe and Broca’s area, which is involved in language (Haier et al., 2005), whereas in men it correlates with the volume of the frontal and parietal lobes; suggesting that men and women use different brain areas to achieve a similar IQ. Scarce literature has analyzed cognitive performance in women in respect to their menstrual cycle phase or hormonal status. In the few instances that the menstrual cycle phase was the primary research aim, typically the research focused on cognitive domains, using mental rotation or language tasks (Masters and Sanders, 1993; Fernández et al., 2003; Bell et al., 2006; Frings et al., 2006; Schoning et al., 2007; Pletzer et al., 2011). Women in the early follicular phase were inferior to men at a task requiring response inhibition to obvious versus less obvious stimuli; however, no sex differences in neural activation were associated with different performance levels (Halari and Kumari, 2005). A similar study determined that sex differences in performance on verbal and spatial cognitive tasks were not significantly related to endogenous hormone levels in men and women during the early follicular phase of the menstrual cycle (Halari et al., 2005).

### Hormonal impact on brain cognitive performance

Both androgens and estrogens have been shown to influence the organization of neural structure and function (Miodovnik et al., 2012). The prenatal hormonal levels influences the development of brain structures involved not only in sexual behaviors but also in cognition, memory, aggression and mood, resulting in a multitude of phenotypes that vary both within and between the sexes (McCarthy et al., 2009). The mechanisms underlying the sexual differentiation of the brain, however, are complex and incompletely understood. Sex steroids may act directly on sexually dimorphic regions of the brain; they may affect the spatial patterning of sex steroid receptors across brain regions; or they may impact the pituitary-gonadal axis, i.e., negative feedback from excess estradiol would result in decreased gonadotropin release and, subsequently, diminished testosterone serum levels (Rubinow and Schmidt, 1996; Miodovnik et al., 2012).

Previous studies have explored the link between sex hormones and other female-related mood disorders such as premenstrual dysphoric disorder (PMDD), (unipolar) postpartum depression, perimenopausal depression, and bipolar disorder (Schmidt et al., 1998; Bloch et al., 2000; Frey and Dias, 2014).

Several (not many) studies have investigated the impact of fluctuating sex hormone levels during the menstrual cycle on the interplay between emotion and cognition in healthy regularly cycling women. This lack of knowledge is remarkable, considering the evidence for major emotional disorders occurring specifically during normal hormonal swings in the lifespan of women. A recent review of the literature by Sacher et al. (2013) summarized neuroimaging studies that showed that menstrual cycle phase affected the reaction to emotional stimuli and reward, as evidenced by behavioral biases in reaction time and neural activation. In line with this evidence, the menstrual cycle also appeared to impact a neural network implicated in cognitive control of emotion. It was suggested by these authors that the menstrual cycle be considered as a modulating factor when examining the behavioral and neural response to emotional stimuli.

### Sex differences in ADHD as a model for cognitive function

As with many neurodevelopmental disorders, the prevalence of attention deficit/hyperactivity disorder (ADHD) differs in males and females (American Psychiatric Association, 2013). ADHD is considered as a model of neuro-developmental cognitive functions and disorders (Pennington, 2006). Yet, relatively very little is known about the role of sex hormones in the pathophysiology of ADHD, and only recently has ADHD in females become the focus of clinical studies, while most previous research included mainly males (Gross-Tsur et al., 2006; Skogli et al., 2013). Males are at least twice more likely to be identified with ADHD than females (American Psychiatric Association, 2013). Research on gender differences suggests that girls may be consistently under identified and under diagnosed because of differences in the expression of the disorder among boys and girls (Skogli et al., 2013). The precise mechanisms underlying this sex difference are poorly understood and scarcely studied. Genetic and hormonal factors cited as potential causes of the male preponderance in ADHD but other factors, however, may contribute to this disparity (Mahone, 2010). Limitations inherent in the DSM nomenclature may contribute to the under-diagnosis of ADHD in females, rating scales may not adequately capture symptom severity among females, teachers are more likely to refer males than females for treatment for ADHD (Sciutto et al., 2004; Waschbusch and King, 2006). Thus, functional difficulties among females with ADHD may go unrecognized and untreated, and it remains unclear to what extent biological factors (genes, hormones) drive the preponderance of males diagnosed with ADHD (Lemiere et al., 2010; Mahone, 2010). Recent electroencephalogram (EEG) study has demonstrated that girls’ EEG activity failed to replicate differences found previously in mixed-sex groups (Dupuy et al., 2013). The authors concluded that this reinforces the notion that it is no longer appropriate to apply the male-based literature to all ADHD groups, rather, the use of single-sex subject groups is necessary in EEG research of ADHD (Dupuy et al., 2013). Most studies regarding ADHD in females suggest confusing and non-consistent conclusions. Some suggest that ADHD school-age girls have far more impairment than their healthy female peers, with significant deficits in internalizing and externalizing disorders, and greater impairment in academic, social, and family domains (Biederman et al., 1999; Gershon, 2002; Zalecki and Hinshaw, 2004). Others suggest that ADHD in school-age boys and girls is more similar than different (Arcia and Conners, 1998; Castellanos et al., 2002; Yang et al., 2004). None of these studies examined the possible relationship between sex hormones and ADHD symptoms. As reviewed above, there is a growing body of literature showing that sex hormones have the ability to regulate intracellular signaling systems that are thought to be abnormal also in ADHD. Thus, it is conceivable to believe that this functional interaction between sex hormones and molecules involved with synaptic plasticity and neurotransmitter systems may be associated with some of the clinical characteristics of women with ADHD (Frey and Dias, 2014).

The investigation of biological models involving the role of estrogen, progesterone, and other sex steroids has also the potential to generate new and improved diagnostic and treatment strategies that could change the course of cognitive-behavioral disorders such as ADHD in women.

## Summary

Sex differences in brain morphology, function and neurochemistry are likely to impact normal and abnormal behavior and function. Until the role of sex hormones in the female human brain is understood, it is important to take into account critical variables, such as menstrual cycle phase, hormonal status (e.g., post partum, perimenopause, menopause), and external hormonal use (e.g., combined oral contraception, hormonal replacement therapy at menopause). In spite of the impact of sex hormones on major neurotransmitter systems of the brain in a variety of clinical settings, the menstrual cycle is usually entered to statistical analyses as a nuisance regressor (Lonsdorf et al., 2011), or controlled for by only testing male samples (Karama et al., 2011). The menstrual cycle offers a unique opportunity to study whether and how subtle fluctuations of sex hormones and specific combinations of sex hormones influence neuronal circuits implicated in the cognitive regulation of emotional processing.

We suggest that the menstrual cycle should be considered as a modulating factor when examining cognitive response to emotional information in women. Furthermore, with the introduction of sensitive tests to measure cognitive performance and imaging techniques to visualize brain morphology and study its neurochemistry, it is now becoming possible to carefully analyze cognitive performance in women by their menstrual cycle phase, or current hormonal status. This may lead to better understanding of the sex hormone impact on women’s brain in health as well as in ADHD and may resolve the inconsistency of the findings in women with ADHD.
